# Safety and efficacy of Ovaleap® (recombinant human follicle-stimulating hormone) for up to 3 cycles in infertile women using assisted reproductive technology: a phase 3 open-label follow-up to Main Study

**DOI:** 10.1186/s12958-016-0164-y

**Published:** 2016-06-10

**Authors:** Thomas Strowitzki, Waldemar Kuczynski, Arnd Mueller, Peter Bias

**Affiliations:** Department of Gynecological Endocrinology and Reproductive Medicine, University of Heidelberg, Im Neuenheimer Feld 440, 69120 Heidelberg, Germany; Centre for Reproductive Medicine, Cryobank, Poland; Department of Gynaecology and Gynaecological Oncology, Medical University, Bialystok, Poland; Merckle GmbH, 89079 Ulm, Germany

**Keywords:** Ovaleap, Follitropin alfa, Infertility, Assisted reproductive technology (ART), r-hFSH

## Abstract

**Background:**

Ovaleap® (follitropin alfa), a recombinant human follicle-stimulating hormone intended for use in controlled ovarian stimulation in women undergoing assisted reproductive technologies (ART), showed therapeutic equivalence to Gonal-f® in a multinational, multicenter, randomized, controlled, assessor-blind phase 3 Main Study. The current study examined safety, including immunogenicity, and efficacy of Ovaleap® in an open-label, uncontrolled, follow-up treatment period of up to 2 additional treatment cycles in patients who did not become pregnant in the phase 3 Main Study.

**Methods:**

Patients with negative biochemical or clinical pregnancy in the phase 3 Main Study, regardless of treatment group (ie, Ovaleap® or Gonal-f®), were eligible to participate. Patients received Ovaleap® (Merckle Biotec GmbH, Ulm, Germany) for up to 2 additional cycles, administered using a reusable semi-automated pen device. The primary objective was the assessment of safety, including adverse events (AEs), ovarian hyperstimulation syndrome (OHSS), and anti-drug antibodies. Tolerability, patient satisfaction with the Ovaleap® pen device, and efficacy outcomes (as evaluated in the Main Study) were also assessed.

**Results:**

One hundred forty-seven patients were included in cycle 2, and 61 patients were included in cycle 3. In cycles 2 and 3, 10.9 % (16/147) and 6.6 % (4/61) of patients experienced treatment-emergent AEs (TEAEs), respectively. Three serious TEAEs (ie, appendicitis, OHSS, and borderline ovarian tumor) were reported and successfully resolved. The OHSS TEAE was the only OHSS reported in the study (0.7 % [1/147]). Positive findings on anti-drug antibody assays in 6 serum samples did not show neutralizing activity or clinical relevance in biochemical pregnancy rate. No hypersensitivity reaction occurred. Most patients reported “very good”/“good” local tolerability. All patients were “very confident”/“confident” about dose accuracy and correctness of the injection. They all found use of the pen “very convenient”/“convenient” and were all “very satisfied”/“satisfied” with the pen device. Efficacy outcomes were consistent with the phase 3 Main Study.

**Conclusions:**

These findings further support the safety, including immunogenicity, and efficacy of Ovaleap® for stimulation of follicular development in infertile women undergoing ART. The findings support continued use of Ovaleap® for multiple cycles or a switch to Ovaleap® if pregnancy is initially not achieved with Gonal-f®.

**Trial registration:**

EudraCT number: 2009-017674-20. Current controlled trials register number: ISRCTN74772901.

## Background

Recombinant human follicle-stimulating hormone (r-hFSH) provides an ovulation induction or ovarian stimulation treatment option for women with infertility being treated with assisted reproductive technologies (ART). The production of r-hFSH in mammalian cells using recombinant DNA technology, as compared with urinary-derived gonadotropin preparations, allows for greater availability of a biochemically pure FSH preparation, without urinary protein contaminants and with reduced product variability that can be associated with different urinary purification techniques [1, 2]. Additionally, r-hFSH has the potential benefit of reduced risk of immunological reactions due to the absence of impurities [1, 3]. Low immunogenicity also allows for subcutaneous (SC) administration [2].

XM17 (Ovaleap®; follitropin alfa), an r-hFSH manufactured in Chinese hamster ovary cells, is intended for use in controlled ovarian stimulation in women undergoing ART, for treatment of anovulation—including polycystic ovarian syndrome, and for stimulation of spermatogenesis. Ovaleap® (Merckle Biotec GmbH, Ulm, Germany), approved by the European Medicines Agency in 2013 [4], was developed as a biosimilar (ie, this agent demonstrated similarity in physicochemical characteristics, efficacy, and safety [5]) to Gonal-f®, following clinical development guidelines established by the Committee for Medicinal Products for Human Use [6]. Two pharmacokinetic studies of Ovaleap® conducted in healthy female subjects demonstrated dose-proportionality and bioequivalence to Gonal-f®, as indicated by peak plasma concentration (C_max_) and area under the concentration-time curve (AUC_0-t_) [7, 8]. A multinational, multicenter, randomized, controlled, assessor-blind phase 3 patient-study—henceforth to be termed “Main Study”—compared efficacy and safety endpoints of Ovaleap® and Gonal-f® in infertile women using ART and demonstrated therapeutic equivalence [9]. The mean ± SD numbers of oocytes retrieved (primary efficacy endpoint) were 12.2 ± 6.7 for Ovaleap® and 12.1 ± 6.7 for Gonal-f®. Treatment-emergent adverse event (TEAE) profiles were similar in patients receiving Ovaleap® and Gonal-f®.

The current study covers an open-label, uncontrolled, follow-up treatment period to the Main Study. The open-label treatment period consisted of up to 2 additional Ovaleap® treatment cycles following the Main Study for women who did not become pregnant in the Main Study. The open-label follow-up study’s primary objective was to assess Ovaleap® safety, including immunogenicity, during second and third treatment cycles and following the switch from Gonal-f® in the Main Study. Secondary objectives were to assess number of oocytes retrieved and total Ovaleap® dose.

## Methods

### Patient population

Infertile but otherwise healthy, normal gonadotropic women with regular menstrual cycles, aged 18 to 37 years, were eligible to participate in the multinational, multicenter, randomized, controlled, assessor-blinded, parallel-group phase 3 Main Study that compared Ovaleap® and Gonal-f® in a long gonadotropin-releasing hormone (GnRH) agonist protocol [9]. Study inclusion and exclusion criteria for the phase 3 Main Study have been previously described (Appendix 1). Patients who had a negative biochemical or clinical pregnancy following the phase 3 Main Study, regardless of their treatment group (ie, Ovaleap® or Gonal-f®), were eligible to participate in the follow-up study. The open-label follow-up study included patients from 19 centers in 5 countries.

### Study design

**Fig. 1:**
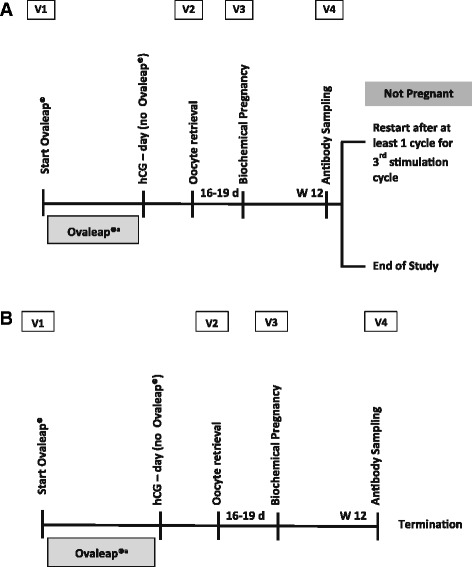
Study Design Showing Cycle 2 (Panel A) and Cycle 3 (Panel B) of Ovaleap® Treatment. **a** Ovaleap® Cycle 2. **b** Ovaleap® Cycle 3. d, days; hCG, human chorionic gonadotropin; V1, Visit 1; V2, Visit 2; V3, Visit 3; V4, Visit 4; W 12, Week 12. ^a^The duration of Ovaleap® treatment was at the discretion of the investigator

The study protocol and informed consent documents were approved by the relevant independent ethics committees. The study was conducted in accordance with the Good Clinical Practice Consolidated Guideline, according to the International Conference on Harmonisation and the Declaration of Helsinki (1996). Regardless of their r-hFSH treatment in the Main Study, all patients in the open-label follow-up study received Ovaleap® for up to 2 additional cycles following completion of the Main Study (Fig. 1). Prior to participation in the open-label follow-up study, at least 1 spontaneous cycle was required between the negative biochemical or clinical pregnancy in the Main Study and the start of cycle 2, the second ovarian stimulation cycle for the patient. The same applied for the third ovarian stimulation cycle (cycle 3). Throughout the open-label follow-up study, the investigator and the embryologist remained blinded to each patient’s assignment of randomized study drug in the Main Study.

Cycle 2 and cycle 3 of Ovaleap® treatment each included 4 study visits, and treatment was started at Visit 1. Pituitary down-regulation was performed at the discretion of the investigator, using GnRH agonists, including busereline, nafarelin, goserelin, and triptorelin (cycle 2 = 68.7 % [101/147] of patients; cycle 3 = 70.5 % [43/61] of patients) or GnRH antagonists, including cetrorelix and ganirelix (cycle 2 = 27.2 % [40/147] of patients; cycle 3 = 27.9 % [17/61] of patients). Final maturation of oocytes was triggered by human chorionic gonadotropin (hCG) injection. Vaginal ultrasound examination was performed to count the number of follicles prior to hCG administration. hCG was withheld if there was a risk of OHSS, as indicated by estradiol level >5500 pg/mL and/or >40 follicles on ultrasound. Oocyte retrieval was performed at Visit 2, 34 to 37 h after hCG administration, and in vitro fertilization (IVF) and/or intra-cytoplasmic sperm injection (ICSI) was completed. Outcome categorization (without pronucleus [PN], 1 PN, 2 PNs, or ≥3 PNs) was assessed at 16 to 20 h after incubation. At this time, oocyte quality also was assessed using the Z score [10]. The morphology of the 2 PN oocytes was graded Z1 (best quality) to Z4 (worst quality), based on the number, size, distribution, and alignment of nuclei in the PN. Embryo transfer occurred at the discretion of the embryologist and investigator. At Visit 3, approximately 16 to 19 days following oocyte retrieval, biochemical pregnancy testing (β-hCG test) and end-of-study assessments were completed. For some patients who did not become pregnant in cycle 2, Visit 4 of cycle 2 was performed at the same time as Visit 1 of cycle 3.

Patients were treated individually at the discretion of the investigator. The starting dose of Ovaleap® was determined by the investigator, with the limitation that doses above 450 IU/day were not recommended. The study investigator determined the need for dose adjustments (up or down) to achieve adequate follicular development, based on clinical aspects, serum estradiol levels, and/or ultrasound examinations. Dose adjustments were allowed every 3 to 5 days through Day 20 in increments or multiples of 37.5 IU (but no more than 150 IU) to a maximum of 450 IU/day. The treatment phase was up to 20 days per cycle. The investigator selected the drugs and regimen for pituitary down-regulation, the hCG for ovulation induction, and any luteal support. The first dose of Ovaleap® was administered at the investigator’s study site, and subsequent daily doses were self-administered by subjects. Ovaleap® was supplied in glass cartridges containing 900 IU in 1.5 mL solution and was administered SC from a reusable semi-automated pen device.

### Safety and immunogenicity assessments

The primary objective of the open-label follow-up study was the assessment of safety, including immunogenicity. Assessments included AEs, OHSS, overall and local tolerability, laboratory variables (ie, clinical chemistry, hematology, and endocrinology), vital signs (ie, blood pressure and heart rate), clinical picture, and immunogenicity via antibody testing. All AEs were considered treatment-emergent AEs and were defined as any unfavorable or unintended sign, symptom, or disease temporally associated with the use of Ovaleap®, whether or not it was considered related to the use of Ovaleap®. Serious TEAEs were defined as AEs that resulted in death, were life-threatening, required in-patient hospitalization or prolongation of existing hospitalization, resulted in persistent or significant disability/incapacity, were a congenital anomaly/birth defect, or were judged an important medical event that may have jeopardized the patient or may have required medical intervention to prevent one of the listed serious TEAE criteria. Intensity was assessed for both serious and non-serious AEs as mild, moderate, or severe. The investigator assessed the causal relationship of the AE to Ovaleap® as probable, possible, unlikely, not classifiable, or not related. The relationship of treatment-emergent adverse drug reactions (TEADRs) to Ovaleap® was assessed by the investigator as probable, possible, unlikely, not classifiable, or missing. AEs of special interest included OHSS, ectopic pregnancy, pregnancy loss, and embolic and thrombotic events. OHSS severity was defined as moderate, severe, or life-threatening, using criteria from Papanikolaou and Navot [11, 12]. AEs were documented from the time of study consent until 30 days after the last Ovaleap® administration.

Immunogenicity blood samples were obtained for anti-drug antibody testing before Ovaleap® administration, on the day of oocyte retrieval, and 3 months after the final Ovaleap® administration, regardless of the number of cycles. Serum samples were assessed for the presence of both anti-FSH antibodies and anti-N-glycolylneuraminic acid (anti-Neu5Gc) antibodies using validated assays at a central laboratory. The assay detected antibodies against the whole Ovaleap® molecule as well as protein moiety and Neu5Gc moiety. Biological relevance was assessed through a cell-based antibody assay to detect neutralizing reactivity. All assays were fully validated in bioanalytical laboratories.

Overall tolerability was assessed by patients and investigators at the end-of-study visit using a 5-point verbal rating scale (1 = very good, 2 = good, 3 = moderate, 4 = poor, 5 = very poor). Patients assessed their local tolerability (ie, injection-site pain and reactions) to Ovaleap® administration using daily diaries. Injection-site pain was rated from 0 (no pain at all) to 10 (the most severe pain) following each injection. Patients recorded the presence of any injection-site reactions of redness, bruising, swelling, burning, or skin irritation, and they rated the intensity as no reaction, mild, moderate, or severe. Possibly clinically significant laboratory values were defined as >3 times the upper limit or <1/3 the lower limit of the reference range.

### Pen device satisfaction

Patient satisfaction with the Ovaleap® reusable semi-automated pen device was examined using a 7-question questionnaire based on Somkuti (2006) [13]. Subjects rated their confidence in accurately preparing the daily dose (scale: 1 = very confident, 2 = confident, 3 = not confident); their confidence in injecting the correct daily dose (scale: 1 = very confident, 2 = confident, 3 = not confident); the ease of understanding the written instructions (scale: 1 = very easy, 2 = easy, 3 = not easy); the number of times they asked the doctor’s office to explain the administration of the daily dose (>4 times, 4 times, 3 times, twice, once, never); the overall ease of use of the pen (scale: 1 [easiest] to 10 [most difficult]); the convenience of administering study medication with the pen (scale: 1 = very convenient, 2 = convenient, 3 = not convenient); and their satisfaction with administering study medication with the pen (scale: 1 = very satisfied, 2 = satisfied, 3 = not satisfied).

### Secondary endpoints

The total Ovaleap® dose and duration of stimulation; number of follicles >14 mm; number of follicles ≤10 mm, >10–14 mm, >14–17 mm, and >17 mm on the day of hCG administration; cancellation rate (ie, premature study end due to no oocytes retrieved); number of oocytes retrieved; oocyte quality; biochemical pregnancy rate; and ongoing pregnancy rate were examined. The follicle size was determined as the mean of 2 diameters measured on the perpendicular axes in the largest sagittal plane of the follicle. The biochemical pregnancy rate was calculated per started cycle, per oocyte retrieval in the cycle, and per embryo transfer in the cycle. The ongoing pregnancy rate was calculated per embryo transferred as the following: the number of pregnant patients divided by the number of patients who had an embryo transfer × 100.

### Data analysis

The planned open-label follow-up study analyses were descriptive (eg, mean ± SD, median, range, percentages) and exploratory. These analyses included all patients who received at least 1 dose of Ovaleap® during the open-label follow-up study. Analyses were conducted separately for cycle 2 and cycle 3 and for the combined cycles. Results are reported according to the treatment received in the phase 3 Main Study (Ovaleap® versus Gonal-f® patient groups) and overall. SAS version 9.2 was used for all analyses.

## Results

**Fig. 2:**
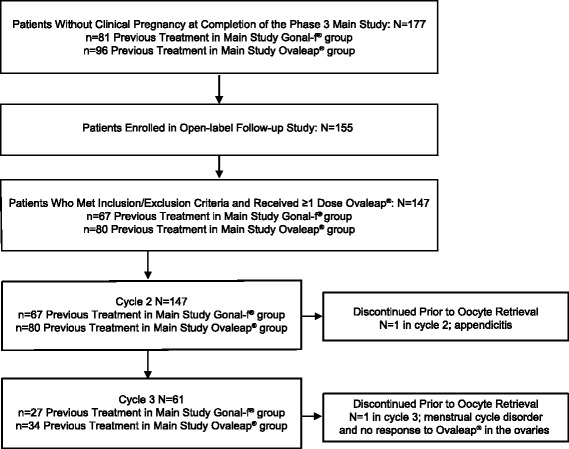
Patient Disposition

One hundred and seventy-seven patients from the phase 3 Main Study did not have a clinical pregnancy and were eligible for enrollment (Fig. 2). A total of 155 patients were enrolled, and 147 patients met inclusion/exclusion criteria and received at least 1 dose of Ovaleap®. Cycle 2 included 147 patients, and cycle 3 included 61 patients from study centers across 5 countries (Appendix 2). Demographic characteristics were comparable between the patients who had received Ovaleap® versus Gonal-f® during the phase 3 Main Study in both cycle 2 and cycle 3 of the open-label follow-up study (Table 1).

**Table 1:** Demographic Characteristics^a^

		Previous Treatment in Main Study	
	Total Group	Ovaleap®	Gonal-f®
Cycle 2	*N* = 147	*N* = 80	*N* = 67
Age, years			
Mean (SD)	31.6 (3.3)	31.4 (3.4)	31.9 (3.0)
Age, *n* (%)			
< 30 years	37 (25.2)	23 (28.8)	14 (20.9)
30 to 34 years	77 (52.4)	42 (52.5)	35 (52.2)
> 34 years	33 (22.4)	15 (18.8)	18 (26.9)
Weight, kg			
Mean (SD)	63.2 (9.4)	63.1 (9.8)	63.4 (8.8)
BMI, kg/m^2^			
Mean (SD)	22.7 (2.8)	22.7 (2.8)	22.6 (2.8)
Smoker, *n* (%)	20 (13.6)	13 (16.3)	7 (10.4)
Alcohol consumption, *n* (%)	25 (17.0)	18 (22.5)	7 (10.4)
Cycle 3	*N* = 61	*N* = 34	*N* = 27
Age, years			
Mean (SD)	31.6 (3.2)	31.1 (3.5)	32.3 (2.8)
Age, *n* (%)			
< 30 years	13 (21.3)	10 (29.4)	3 (11.1)
30 to 34 years	34 (55.7)	19 (55.9)	15 (55.6)
> 34 years	14 (23.0)	5 (14.7)	9 (33.3)
Weight, kg			
Mean (SD)	63.5 (8.9)	62.5 (8.2)	64.7 (9.7)
BMI, kg/m^2^			
Mean (SD)	23.0 (2.9)	22.8 (2.8)	23.3 (3.1)
Smoker, *n* (%)	11 (18.0)	7 (20.6)	4 (14.8)
Alcohol consumption, *n* (%)	13 (21.3)	8 (23.5)	5 (18.5)

*BMI* body mass index; *SD* standard deviation

^a^Reasons for and duration of infertility were comparable between the Ovaleap® and Gonal-f® groups in the Main Study [9]

### Safety and immunogenicity

In the Main Study, the overall TEAE frequency was 47/299 (15.7 %) patients. None of the patients who entered the open-label follow-up study had ongoing AEs from the Main Study. The frequency of TEAEs was low after treatment in cycle 2 and cycle 3 (Appendix 3); 16/147 (10.9 %) patients experienced TEAEs in cycle 2, 4/61 (6.6 %) patients experienced TEAEs in cycle 3, and 19/147 (12.9 %) patients experienced TEAEs in the combined cycles 2 and 3. Only 1 patient experienced TEAEs in both cycle 2 and cycle 3. In cycles 2 and 3 combined, the overall frequencies of TEAEs were comparable in patients previously treated with Ovaleap® versus Gonal-f® in the Main Study (11.3 % versus 14.9 %, respectively). All TEAEs resolved.

Among TEAEs categorized as non-serious, back pain rated as severe intensity was reported in 1 patient in cycle 2. Two TEAEs were categorized by the investigator as TEADRs probably related to Ovaleap®: 1 mild injection-site erythema, pruritus, and hematoma and 1 mild lower abdominal pain. Three serious TEAEs were reported: 1 appendicitis, 1 OHSS, and 1 borderline ovarian tumor. The appendicitis was assessed as unrelated to study medication and resolved but resulted in premature study discontinuation for the patient. The OHSS occurred in cycle 2 and led to hospitalization. This was the only OHSS reported in the follow-up study (0.7 % [1/147]). It was assessed as a TEADR probably related to study medication and resolved. The borderline ovarian tumor experienced by 1 patient was diagnosed during planned laparoscopy, a standard procedure following 2 unsuccessful IVF procedures. It was assessed as a TEADR unlikely related to study medication. The AE outcome was reported as recovered with right ovariectomy and salpingectomy, left partial salpingectomy, omentectomy, and appendectomy. There were no deaths or other serious or significant AEs and no ectopic pregnancy, pregnancy loss, or embolic or thrombotic events.

A total of 485 blood samples from 147 patients participating in the open-label follow-up study were analyzed for immunogenicity. Only 6 patients had confirmed positive findings against Ovaleap® and Neu5Gc during cycle 2. Four patients had pre-existing anti-drug antibodies against Neu5Gc in cycle 2, and the remaining 2 patients developed antibodies against Neu5Gc. None of the samples showed neutralizing activity; therefore, no patient developed neutralizing antibodies. Biochemical pregnancy rates were comparable between patients who had antibodies against Ovaleap®, which were mainly against Neu5Gc epitope, and patients who did not, indicating the positive findings were not clinically relevant. None of the patients showed a hypersensitivity reaction during the study.

Potentially clinically significant laboratory values were obtained in 2 patients, including below reference range alanine aminotransferase values during cycle 3 in 1 patient and depressed basophil values at the start of cycle 2 for 1 patient. No TEAEs related to vital signs were reported, and results for laboratory safety variables and vital signs did not raise any safety concerns.

### Tolerability

All patients rated Ovaleap® overall tolerability as “very good” or “good.” Investigators rated tolerability as “very good” or “good” in all but 4 patients who were rated as showing moderate tolerability.

Patient reports of local tolerability included ratings of injection-site pain (scale ranging from 0 [no pain at all] to 10 [most severe pain]) at each treatment day. Apart from 1 patient who reported a rating of 6 on a given treatment day, mean scores were low, being not more than 1 on all but 2 treatment days (respectively, 1.1 and 1.7). In cycle 2, most patients (>80 %) did not report any injection-site reactions (ie, bruising, burning, redness, skin irritation, and swelling) after the first injection. Across all injections in cycle 2, moderate reactions were reported in 4 patients and a severe reaction was only reported for bruising in 1 patient on 1 stimulation day. In cycle 3, mild reactions were reported in 11 patients and moderate reactions in 3 patients. Severe injection-site reactions were reported in 7 patients on a single stimulation day.

### Satisfaction with pen device

In cycle 2, the mean rating for overall ease of use of the Ovaleap® injection pen was 1.4 (scale ranging from 1 [easiest] to 10 [most difficult]) (Table 2). All patients were “very confident” or “confident” about the accuracy of the dose (83.2 % and 16.8 %, respectively) and about correctness of the injection (83.9 % and 16.1 %, respectively). The instructional text for the pen was rated “very easy” to understand by 75.5 % (108/143) of patients and “easy” by 23.8 % (34/143) of patients. The frequency of additional explanation for how to use the pen was reported as “never” or “one time” by 98.6 % (141/143) of patients. All patients found the pen “very convenient” or “convenient” (76.9 and 23.1 %, respectively) and were “very satisfied” or “satisfied” (83.2 and 16.8 %, respectively) with the pen. In cycle 3, results were slightly more favorable than for cycle 2, which can be expected due to increasing familiarization with the injection pen (data not shown).

**Table 2:** Patient Satisfaction With the Ovaleap® Pen at the End of Cycle 2

		Previous Treatment in Main Study	
Question	Total Group	Ovaleap®	Gonal-f®
	*N* = 143	*N* = 77	*N* = 66
	*n* (%)	*n* (%)	*n* (%)
Overall ease of use^a^			
Mean (SD)	1.4 (1.1)	1.2 (0.5)	1.5 (1.4)
Confidence about accurate dose			
Very confident	119 (83.2)	66 (85.7)	53 (80.3)
Confident	24 (16.8)	11 (14.3)	13 (19.7)
Not confident	0	0	0
Confidence about correct injection			
Very confident	120 (83.9)	67 (87.0)	53 (80.3)
Confident	23 (16.1)	10 (13.0)	13 (19.7)
Not confident	0	0	0
Plainness of instructional text			
Very easy	108 (75.5)	58 (75.3)	50 (75.8)
Easy	34 (23.8)	19 (24.7)	15 (22.7)
Not easy	1 (0.7)	0	1 (1.5)
Frequency of explanation of administration			
Never	87 (60.8)	52 (67.5)	35 (53.0)
Once	54 (37.8)	25 (32.5)	29 (43.9)
Twice	2 (1.4)	0	2 (3.0)
Convenience of pen usage			
Very convenient	110 (76.9)	56 (72.7)	54 (81.8)
Convenient	33 (23.1)	21 (27.3)	12 (18.2)
Not convenient	0	0	0
Satisfaction with administration			
Very satisfied	119 (83.2)	63 (81.8)	56 (84.8)
Satisfied	24 (16.8)	14 (18.2)	10 (15.2)
Not satisfied	0	0	0

^a^The overall ease of use of the pen was rated using a scale ranging from 1 (easiest) to 10 (most difficult)

### Secondary endpoints

The mean ± SD (median) total dose of Ovaleap® in cycle 2 was 1998 ± 771 (1875) IU, and in cycle 3, it was 2113 ± 939 (1875) IU; the median treatment duration for both cycles 2 and 3 was 10 days (Table 3). The mean ± SD number of follicles >14 mm on the hCG injection day in cycle 2 was 10.0 ± 5.6 and in cycle 3 was 11.9 ± 5.4 (Appendix 4). At least 1 oocyte was harvested in 99.3 % (146/147) of patients in cycle 2 and 98.4 % (60/61) of patients in cycle 3. The mean ± SD (median [range]) number of oocytes was 12.1 ± 5.9 (11.0 [2.0 to 36.0]) in cycle 2 and 13.5 ± 6.5 (12.0 [3.0 to 33.0]) in cycle 3 (Table 3). Oocyte quality was similar to that observed in the Main Study (Appendix 5).

**Table 3:** Total Ovaleap® Dose and Duration of Stimulation and Number of Oocytes Retrieved

		Previous Treatment in Main Study	
	Total Group	Ovaleap®	Gonal-f®
Cycle 2	*N* = 147	*N* = 80	*N* = 67
Total Ovaleap® dose, IU			
Mean (SD)	1998 (771)	1925 (706)	2086 (840)
Median (range)	1875 (450–4313)	1763 (1013–3750)	2025 (450–4313)
Duration of Ovaleap® stimulation, days			
Mean (SD)	9.9 (1.8)	9.7 (1.8)	10.0 (1.9)
Median (range)	10.0 (4.0–14.0)	10.0 (5.0–14.0)	10.0 (4.0–14.0)
Number of oocytes retrieved^a^			
Mean (SD)	12.1 (5.9)	12.0 (5.8)	12.1 (6.0)
Median (range)	11.0 (2.0–36.0)	11.0 (3.0–29.0)	11.0 (2.0–36.0)
Cycle 3	*N* = 61	*N* = 34	*N* = 27
Total Ovaleap® dose, IU			
Mean (SD)	2113 (939)	1883 (687)	2402 (1132)
Median (range)	1875 (750–5400)	1800 (750–3450)	2100 (788–5400)
Duration of Ovaleap® stimulation, days			
Mean (SD)	9.9 (1.7)	9.6 (1.7)	10.3 (1.7)
Median (range)	10.0 (5.0–14.0)	9.0 (5.0–13.0)	10.0 (7.0–14.0)
Number of oocytes retrieved^b^			
Mean (SD)	13.5 (6.5)	12.3 (6.2)	15.0 (6.5)
Median (range)	12.0 (3.0–33.0)	11.0 (3.0–30.0)	12.0 (6.0–33.0)

*SD* standard deviation

^a^Total Group *N* = 146; Ovaleap® *N* = 80; Gonal-f® *N* = 66

^b^Total Group *N* = 60; Ovaleap® *N* = 33; Gonal-f® *N* = 27

The median number of embryos obtained per patient was 3 (range 0 to 19) in cycle 2 and 4 (range 2 to 15) in cycle 3. The median number of embryos transferred in cycle 2 was 2 (range 0 to 4) and in cycle 3 was 2 (range 1 to 4). Biochemical pregnancy rates, based on the β-hCG test, were 31 % during both cycle 2 and cycle 3 (46/147 and 19/61, respectively), and the overall rate for combined cycle 2 and 3 was 42.9 % (63/147) (Table 4). Two of the patients with biochemical pregnancy in cycle 2 did not have clinical pregnancy and went on to be treated with Ovaleap® in cycle 3. Including only those patients with embryo transfer, the ongoing pregnancy rate in cycle 2 was 25.9 % (37/143), in cycle 3 was 21.7 % (13/60), and in combined cycles 2 and 3 was 34.7 % (50/144).

**Table 4:** Pregnancy rates

		Previous Treatment in Main Study	
	Total Group	Ovaleap®	Gonal-f®
Cycle 2			
Biochemical pregnancy, *n*/*N* (%)	46/147 (31.0)	24/80 (30.0)	22/67 (32.8)
Ongoing pregnancy^a^, *n*/*N* (%)	37/143 (25.9)	21/79 (26.6)	16/64 (25.0)
Cycle 3			
Biochemical pregnancy, *n*/*N* (%)	19/61 (31.0)	10/34 (29.4)	9/27 (33.3)
Ongoing pregnancy^a^, *n*/*N* (%)	13/60 (21.7)	8/33 (24.2)	5/27 (18.5)
Combined Cycles 2 and 3			
Biochemical pregnancy, *n*/*N* (%)	63/147 (42.9)	33/80 (41.3)	30/67 (44.8)
Ongoing pregnancy^a^, *n*/*N* (%)	50/144 (34.7)	29/80 (36.3)	21/64 (32.8)

^a^ *N* includes only those patients with embryo transfer

In cycle 2, one patient terminated the study early due to the AE appendicitis, resulting in an overall cancellation rate prior to oocyte retrieval of 0.7 % (1/147). In cycle 3, one patient terminated the study early due to menstrual cycle disorders and no response to Ovaleap®, resulting in an overall cancellation rate of 1.6 % (1/61).

## Discussion

The safety and efficacy findings in this open-label, non-controlled single therapy follow-up study involving up to 2 additional Ovaleap® treatment cycles beyond the Main Study were comparable to the outcomes in the Main Study, which was a multinational, multicenter, randomized, controlled, assessor-blind phase 3 comparison of Ovaleap® and Gonal-f® in patient groups of infertile women using ART [9]. Overall frequencies of TEAEs in combined cycle 2 and cycle 3 in the open-label follow-up study were low and comparable between patient groups treated with Ovaleap® or Gonal-f® in the phase 3 Main Study. There were no clinically relevant differences in safety results and no new or unexpected safety-related findings. The positive findings of anti-drug antibodies against Ovaleap®, which were characterized as low-titer and predominantly pre-existing Neu5Gc-specific antibodies, lacked clinical relevance. The samples did not show neutralizing activity, patients did not differ in rates of biochemical pregnancy, and no hypersensitivity reactions occurred. An overview of the highly sensitive immunogenicity assays and outcomes in the Main Study and the follow-up study indicated lack of clinical relevance [14]. The mean number of oocytes retrieved and oocyte quality in the current study were similar to the Main Study [9] and were comparable to those in prior studies of Gonal-f® [15–20]. Additionally, a greater percentage of patients reported satisfaction with the Ovaleap® injection pen device than those that have been reported in studies of satisfaction and preference with other pen devices (100 % versus 80 % and 84 %, respectively) [21, 22]. Altogether, the study findings, including examination of immunogenicity, support the safety and efficacy of a switch to Ovaleap® if pregnancy is initially not achieved with Gonal-f® and suggest a course of up to 3 cycles of treatment with Ovaleap® is safe and well tolerated by patients.

Total dose of r-hFSH (Ovaleap® or Gonal-f®) increased from the first treatment cycle in the Main study to the second and third Ovaleap® cycles in the current study. This is consistent with previous studies showing increased gonadotropin dose in patients receiving multiple IVF treatment cycles [23, 24] and can be understood as being due to the poorer response of these patients to FSH stimulation, as indicated by not achieving pregnancy following the first IVF cycle. However, the median total dose of Ovaleap® in the current study was consistent with the Gonal-f® dose used in other clinical trials for optimal follicular development [15, 17–19]. Likewise, the biochemical and ongoing pregnancy rates in the current study were expected to be slightly lower than those found in the Main Study as these patients did not become pregnant in the initial IVF treatment cycle (ie, in the Main study).

The availability of new ovarian stimulation treatment options that are therapeutically equivalent in terms of efficacy and safety to existing treatments, with improved use convenience and high levels of treatment-related patient satisfaction, may meet an important need among infertile women using ART [2]. Patient perceptions of their ovarian stimulation treatment experience related to ease of use and reduced dose variability have been shown to be important in determining patient treatment preference [25]. Additionally, use of pen devices and simplification of injection procedures may be important in determining patient treatment satisfaction and preference [26]. Consistent with these treatment needs, therapeutic equivalence has been shown between Ovaleap® and Gonal-f® in the Main Study, and patients have reported high satisfaction and confidence in the dose preparation and injection experience with the Ovaleap® pen device and rated pen use as very convenient/convenient. These findings support Ovaleap® as an important addition to the ovarian stimulation treatment options for infertile women using ART.

Limitations of the current study include the open-label, uncontrolled study design; however, throughout the follow-up study, the investigator and embryologist remained blinded to the r-hFSH treatment received by patients in the Main Study. Additionally, the study analyses were descriptive and exploratory, with no formal statistical tests planned or completed. Generalization of the current study outcomes to other patient populations should consider the leanness of the included patients, as indicated by the mean body mass index of 22 to 23 kg/m^2^. Additionally, further research is necessary for evaluation of the safety and efficacy of multiple treatment cycles of Ovaleap® in ART, especially in older women.

## Conclusions

Ovaleap® showed a favorable safety and tolerability profile, comparable with Gonal-f®, following second and third treatment cycles. There were no new or unexpected safety findings. Efficacy endpoints in the open-label follow-up study were consistent with those found in the phase 3 randomized, controlled, assessor-blinded, parallel-group Main Study. Level of satisfaction with the Ovaleap® pen was very high. The findings further support the safety and efficacy of Ovaleap® for stimulation of follicular development in infertile women undergoing ART, and they support continued use of Ovaleap® for multiple cycles and a switch from Gonal-f® to Ovaleap® if pregnancy is not achieved following the initial cycle.

## Appendix 1

**Table 5:** Main Study Inclusion and Exclusion Criteria for Selection of Study Population

Inclusion Criteria
▪ Infertile but otherwise healthy women aged 18 to 37 years of any racial origin
▪ Normal gonadotropic women with regular menstrual cycles of 21–35 days
▪ 2 confirmed normal ovaries and undergoing superovulation for ART
▪ BMI ≥18 kg/m^2^ and ≤29 kg/m^2^
▪ Basal FSH, estradiol, prolactin, and thyroid-stimulating hormone concentrations in the normal range
Exclusion Criteria
▪ History of more than 2 previously completed consecutive unsuccessful in vitro fertilization cycles; >3 miscarriages; history of a severe OHSS; primary ovarian failure or being categorized as poor responders; hypersensitivity or allergy to r-hFSH preparations; or neoplasm or history of chemotherapy or radiation therapy
▪ Malformation of sexual organs incompatible with pregnancy or one or both ovaries inaccessible for oocyte retrieval
▪ Administration of clomiphene or gonadotropins within 30 days prior to enrollment
▪ Any significant cardiovascular, pulmonary, neurologic, endocrine, hepatic, or renal disease
▪ After down-regulation prior to first administration of r-hFSH: serum estradiol ≥50 pg/mL, ovarian cysts >10 mm (verified by ultrasound), or a positive pregnancy test

*ART* assisted reproduction technology; *BMI* body mass index; *FSH* follicle-stimulating hormone; *OHSS* ovarian hyperstimulation syndrome; *r-hFSH* recombinant human follicle-stimulating hormone

## Appendix 2

**Table 6:** Distribution of Patients by Country

		Previous Treatment in Main Study	
	Total Group	Ovaleap®	Gonal-f®
Cycle 2	*N* = 147	*N* = 80	*N* = 67
		*n* (%)	*n* (%)
Belgium + Germany	24 (16.3)	11 (13.8)	13 (19.4)
Czech Republic	28 (19.0)	16 (20.0)	12 (17.9)
Hungary	39 (26.5)	22 (27.5)	17 (25.4)
Poland	56 (38.1)	31 (38.8)	25 (37.3)
Cycle 3	*N* = 61	*N* = 34	*N* = 27
		*n* (%)	*n* (%)
Belgium + Germany	3 (4.9)	1 (2.9)	2 (7.4)
Czech Republic	8 (13.1)	6 (17.6)	2 (7.4)
Hungary	20 (32.8)	12 (35.3)	8 (29.6)
Poland	30 (49.2)	15 (44.1)	15 (55.6)

## Appendix 3

**Table 7:** Treatment-Emergent Adverse Events in Ovaleap® Cycles 2 and 3 Combined

		Previous Treatment in Main Study	
MedDRA Preferred Term	Total Group	Ovaleap®	Gonal-f®
	*N* = 147	*N* = 80	*N* = 67
	*n* (%)	*n* (%)	*n* (%)
Any TEAE	19 (12.9)	9 (11.3)	10 (14.9)
Abdominal pain lower	1 (0.7)	0	1 (1.5)
Anemia	1 (0.7)	0	1 (1.5)
Appendicitis	1 (0.7)	0	1 (1.5)
Application site erythema	1 (0.7)	1 (1.3)	0
Application site hematoma	1 (0.7)	0	1 (1.5)
Application site pruritus	1 (0.7)	1 (1.3)	0
Back pain	1 (0.7)	1 (1.3)	0
Borderline ovarian tumor	1 (0.7)	1 (1.3)	0
Cyst aspiration	1 (0.7)	0	1 (1.5)
Depressed mood	1 (0.7)	0	1 (1.5)
Influenza-like illness	1 (0.7)	1 (1.3)	0
Injection-site erythema	1 (0.7)	0	1 (1.5)
Injection-site pruritus	1 (0.7)	0	1 (1.5)
Menorrhagia	1 (0.7)	1 (1.3)	0
Metrorrhagia	1 (0.7)	1 (1.3)	0
Muscle spasms	1 (0.7)	1 (1.3)	0
Nausea	1 (0.7)	0	1 (1.5)
OHSS	1 (0.7)	0	1 (1.5)
Procedural pain	1 (0.7)	0	1 (1.5)
Pyrexia	1 (0.7)	1 (1.3)	0
Respiratory tract inflammation	1 (0.7)	0	1 (1.5)
Sinusitis	1 (0.7)	0	1 (1.5)
Upper respiratory tract infection	1 (0.7)	1 (1.3)	0
Vaginal discharge	1 (0.7)	1 (1.3)	0
Vaginal infection	2 (1.4)	2 (2.5)	0
Vulvovaginal pruritus	1 (0.7)	1 (1.3)	0

*OHSS* ovarian hyperstimulation syndrome; *TEAE* treatment-emergent adverse event

## Appendix 4

**Table 8:** Number of Follicles >14 mm on the Day of hCG Injection

		Previous Treatment in Main Study	
	Total Group	Ovaleap®	Gonal-f®
Cycle 2	*N* = 146	*N* = 80	*N* = 66
Number of follicles >14 mm			
Mean (SD)	10.0 (5.6)	9.3 (4.8)	10.9 (6.4)
Median (range)	9.0 (0.0–32.0)	9.0 (0.0–31.0)	10.0 (0.0–32.0)
Cycle 3	*N* = 60	*N* = 33	*N* = 27
Number of follicles >14 mm			
Mean (SD)	11.9 (5.4)	10.8 (4.9)	13.3 (5.8)
Median (range)	11.0 (0.0–26.0)	11.0 (0.0–22.0)	11.0 (7.0–26.0)

*hCG* human chorionic gonadotropin; *SD* standard deviation

## Appendix 5

**Table 9:** Oocyte Quality

		Previous Treatment in Main Study	
	Total Group	Ovaleap®	Gonal-f®
Cycle 2	*N* = 146	*N* = 80	*N* = 66
Oocyte quality, *n* (%) oocytes			
Z1 (Best)	162 (18.4)	87 (17.6)	75 (19.3)
Z2	363 (41.2)	210 (42.6)	153 (39.4)
Z3	234 (26.6)	129 (26.2)	105 (27.1)
Z4 (Worst)	122 (13.8)	67 (13.6)	55 (14.2)
Total 2 PN oocytes	881 (100.0)	493 (100.0)	388 (100.0)
Cycle 3	*N* = 60	*N* = 33	*N* = 27
Oocyte quality, *n* (%) oocytes			
Z1 (Best)	71 (17.4)	34 (15.7)	37 (19.5)
Z2	160 (39.3)	84 (38.7)	76 (40.0)
Z3	127 (31.2)	68 (31.3)	59 (31.1)
Z4 (Worst)	49 (12.0)	31 (14.3)	18 (9.5)
Total 2 PN oocytes	407 (100.0)	217 (100.0)	190 (100.0)

*PN* pronucleus; *Z score* zygote scoring system of Scott et al. [10]

## Abbreviations

ART: assisted reproductive technology
AUC_0-t_: area under the concentration-time curve
C_max_: peak plasma concentration
GnRH: gonadotropin-releasing hormone
hCG: human chorionic gonadotropin
IVF: in vitro fertilization
OHSS: ovarian hyperstimulation syndrome
PN: pronucleus
r-hFSH: recombinant human follicle-stimulating hormone
SC: subcutaneous
TEADR: treatment-emergent adverse drug reaction
TEAE: treatment-emergent adverse event
